# Antibodies against *Coxiella burnetii *and pregnancy outcome during the 2007-2008 Q fever outbreaks in the Netherlands

**DOI:** 10.1186/1471-2334-11-44

**Published:** 2011-02-11

**Authors:** Wim van der Hoek, Jamie CE Meekelenkamp, Alexander CAP Leenders, Nancy Wijers, Daan W Notermans, Chantal WPM Hukkelhoven

**Affiliations:** 1 Centre for Infectious Disease Control, National Institute for Public Health and the Environment, P.O. Box 1, 3720 BA Bilthoven, the Netherlands; 2 Department of Medical Microbiology and Infection Control, Jeroen Bosch Hospital, Tolbrugstraat 11, P.O. Box 90153, 5200 ME, 's Hertogenbosch, the Netherlands; 3 Netherlands Perinatal Registry, P.O. Box 8588, 3503 RN, Utrecht, the Netherlands

## Abstract

**Background:**

Q fever has become a major public health problem in the Netherlands. Infection with *Coxiella burnetii *(Q fever) during pregnancy has resulted in adverse pregnancy outcome in the majority of reported cases. Therefore, we aimed to quantify this risk by examining the earliest periods corresponding to the epidemic in the Netherlands.

**Methods:**

Serum samples that had been collected from the area of highest incidence by an existing national prenatal screening programme and data from the Netherlands Perinatal Registry (PRN) on diagnosis and outcome were used. We performed indirect immunofluorescence assay to detect the presence of IgM and IgG antibodies against *C. burnetii *in the samples. The serological results were analyzed to determine statistical association with recorded pregnancy outcome.

**Results:**

Evaluation of serological results for 1174 women in the PRN indicated that the presence of IgM and IgG antibodies against phase II of *C. burnetii *was not significantly associated with preterm delivery, low birth weight, or several other outcome measures.

**Conclusion:**

The present population-based study showed no evidence of adverse pregnancy outcome among women who had antibodies to *C. burnetii *during early pregnancy.

## Background

Infection with *Coxiella burnetii *(Q fever) has recently become a major public health problem in the Netherlands [1,2]. The annual number of laboratory-confirmed cases increased from 10 in 2006 to 168 in 2007. Since then, the upward trend has continued at an alarming rate (weekly updates on number of cases is available in Dutch at: http://www.rivm.nl/cib/themas/Q-koorts/). The 2008 outbreak, comprised of 1000 cases, was ranked as the largest on record anywhere in the world, but this was surpassed in 2009 when a total of 2356 cases were diagnosed.

International literature suggests that untreated acute Q fever infection during pregnancy may result in adverse pregnancy outcome in up to 81% of cases [3,4]. These outcomes include spontaneous abortion, intra-uterine foetal death and premature delivery, or low birth weight. The risk of developing chronic Q fever infection is especially high for pregnant women [5-7]. An estimated 60% of all (male and female) acute Q fever infections occur without clinical symptoms, and among pregnant women this percentage is even higher [8].

Asymptomatic infections may carry the same risk for adverse pregnancy outcome as symptomatic cases [9]. The high proportion of asymptomatic infections and the high risk for adverse effects has led to recommendations which state that in outbreak situations all pregnant women should be offered serological screening for recent Q fever infection; if found positive, long-term antibiotic treatment is proposed [8]. During the first outbreak in the Netherlands in 2007 an effort was made to identify all women who were pregnant or who had recently delivered in the affected area (total approximate population of 4000). Out of 29 women identified through midwives and obstetricians serving the area, 19 underwent serological testing with an indirect immunofluorescence assay (IFA). None of these women presented with symptoms of Q fever, but serological evidence of a recent infection was found for two and for a past infection in one other [10]. As recommended in the international literature, the two women with recent infection were treated with cotrimoxazole for the duration of their pregnancies [4]. These two women delivered under strict hygiene measures and there were no complications during parturition. PCR tests of birth products were negative for *C. burnetii *DNA.

The number of pregnant women with Q fever infection for whom pregnancy outcome has been reported is very limited (<100 women for all studies combined). Furthermore, most studies have been based upon retrospectively collected cases that do not allow quantification of the risk for an adverse outcome of an infection during pregnancy. The 2008 outbreak in the Netherlands was unique from those that had preceded it in that it was not confined to a small geographic area; this widespread infection pattern raised the question of whether screening of pregnant women for acute Q fever infection was necessary and feasible. The answer is largely dependent on the trade-off between adverse effects from untreated Q fever infections and the detrimental side effects associated with long-term antibiotic treatment during pregnancy. Furthermore, it will be necessary to minimize the potential for administering an unnecessary treatment based on a false positive serological result.

An international meeting conducted in July 2008 by the Centre for Infectious Disease Control and the Health Council of the Netherlands concluded that, considering the prevailing conditions in which cases were emerging over a large geographic area, screening of all pregnant women for recent Q fever infection was not justified but that there was an urgent need for more quantitative data to determine the risk of adverse pregnancy outcome among pregnant women with acute Q fever infection in early pregnancy. The findings would then be provided to health planners and policy makers in order to aid in the development of targeted and effective programmes of countermeasures (available in Dutch at: http://www.gezondheidsraad.nl/nl/adviezen/briefadvies-bijeenkomst-over-q-koorts-nederland). To this end, we designed and carried out a population-based retrospective follow-up study of pregnant women in the Netherlands region with highest incidence of Q fever.

## Methods

### Serology

Presence of antibodies against *C. burnetii *during pregnancy was determined by testing sera that had been routinely collected by the Prenatal Screening for Infectious Diseases and Erythrocyte Immunization (PSIE). This is a screening program that is offered free-of-charge to all pregnant women in the Netherlands around the 12^th ^week of pregnancy. It includes screening for hepatitis B, syphilis, and HIV. Participation in the PSIE is voluntary and reaches around 93%. Under this program, the midwife refers the pregnant woman for collection of a blood sample to occur during a regular antenatal care visit. Samples collected under the PSIE can be analyzed at any microbiology laboratory and the remaining serum is often stored for a period of one year after initial analysis.

We obtained the stored sera from regional laboratories located in the northeast of the province of Noord Brabant, which represented the area with the highest Q fever incidence. These sera had been stored in freezers at -20°C after analysis for hepatitis B, syphilis, and HIV. Sera with collection dates from 20 June 2007 to 31 July 2008 were sent on dry ice to the Regional Laboratory of Medical Microbiology and Infection Control in the Jeroen Bosch Hospital located in 's Hertogenbosch, where they were kept in the freezer at -20°C. When a batch of sera was removed from the freezer and thawed, they were analysed the same day for Q fever serology using an IFA device (Focus Diagnostics, Cypress, CA, USA) to detect the presence of IgG and IgM antibodies. In the analysis the positive serological results were categorised in two categories: (1) the presence of anti-phase II IgM and anti-phase II IgG antibodies with a titre of ≥1:64 or a solitary IgM II ≥1:64, suggesting a possible recent infection; and (2) the presence of both anti-phase I and II IgG antibodies with a titre of ≥1:64 and without IgM being present, suggesting a past infection. The remaining samples were scored as negative for *C. burnetii *infection.

### Pregnancy outcome

Information on pregnancy outcome for each of the samples was obtained from the Netherlands Perinatal Registry (PRN). This database represents the joint efforts of the professional organizations of midwives, gynaecologists, obstetrically-trained general practitioners and paediatricians in the Netherlands. The PRN contains perinatal data from 16 weeks of gestation onwards and includes 96% of all births that occur in the Netherlands (http://www.perinatreg.nl/).

A number of outcome variables were assessed in our study: (1) preterm delivery, defined as gestational age under 37 weeks; (2) low birth weight, defined as <2500 grams; (3) low birth weight for gestational age, defined as <10^th ^percentile, derived from sex-, parity, and ethnic background-specific reference curves [11]; (4) foetal or neonatal mortality, up to 7 days (5) congenital malformation, visible at or shortly after birth; and (6) 5-minute Apgar score <7.

### Confounding variables

Information was also collected on a number of potentially confounding variables known to be associated with pregnancy outcome and for which information was available in the PRN. These included maternal age, parity, plurality, ethnic background, socio-economic status, urbanization, and foetal position. Socio-economic status was estimated from the woman's postal code (4 digits) using the methodology developed by the Netherlands Institute for Social Research [12]. The socio-economic status is based on mean income level, the percentage of households with a low income, the percentage of inhabitants without a paid job and the percentage of households with, on average, a low education residing within a particular postal code area. Ethnic background was classified by the healthcare provider as Dutch or other (largely consisting of ethnic groups from Surinam, Morocco, and Turkey). Urbanization was based on the number of households per km^2 ^per postal code area.

### Sample size calculation

It was assumed that the prevalence of antibodies against *C. burnetii *among pregnant women in the high-risk area would be around 10%. This presumption was based on a limited seroprevalence survey among pregnant women during the 2007 outbreak, which showed recent infection in 16% in the outbreak area, 4% in the surrounding 'medium risk' area, and 1% in the rest of the country [10]. The proportion of newborns with low birth weight (<2500 grams) has been reported to be about 7% in the Netherlands [13]. With this outcome measure, the required sample size to detect a relative risk of two with a power of 80% and a significance level of 5% would be 1630. A slightly smaller sample size would be required for the outcome measure preterm delivery (gestational age <37 weeks), which occurs in 8% of pregnancies. From the outset, this study was expected to have insufficient power to detect an increased risk in foetal and neonatal mortality (perinatal mortality) due to the fact this is a rare event which occurs in only about 1% of pregnancies with gestational age ≥22 weeks [13]. Based on these estimates the target sample size was set at 1800, which was expected to provide sufficient power for the outcome measures of low birth weight and preterm delivery, taking into account that some laboratory results might not be linkable to the PRN.

### Statistical analysis

Differences in frequency of pregnancy and delivery features between the women with positive and negative serology were analyzed in contingency tables with the χ2 test. Probability values <0.05 were considered statistically significant. For each outcome, the strength of its association with positive serology was expressed as a crude odds ratio (OR) with 95% confidence interval (CI). We then adjusted the ORs in a multivariable logistic regression analysis for potential confounders, to show the contribution of the examined feature in relation to the other characteristics. Interaction effects were also examined for each confounder variable and serological status by using logistic regression analysis. Missing values occurred for only 0.7% of all confounders and were imputed once using R software [14,15]. The other analyses were conducted using software packages from SAS (version 9.1; SAS Institute, Cary, NC, USA), SPSS (version 17.0; SPSS Inc. Chicago, IL, USA), and Microsoft Excel.

### Ethical considerations

The study was approved by the Medical Research Ethics Committee of the University Medical Centre Utrecht and the Board of the PRN. The latter included approval obtained upon assessment by a privacy commission.

## Results

### Linkage

**Figure 1:**
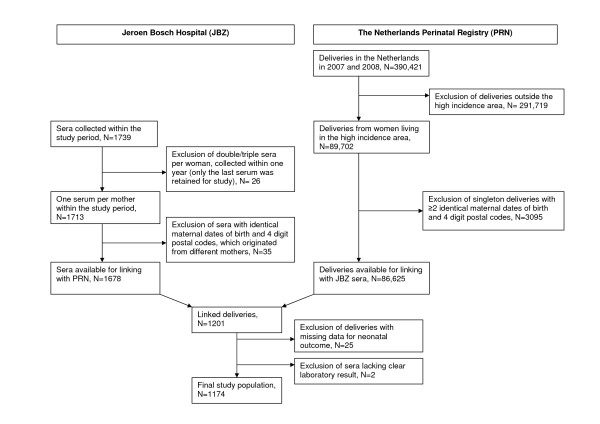
**Flowchart of serological analyses and pregnancy outcome**.

As the analysis was carried out using anonymized samples, without a unique personal identifier, the laboratory results had to be linked to the 2007 and 2008 PRN data by use of limited postal code information (4 digits) and maternal date of birth. Of the 1739 sera samples collected within the study period, 1678 were available for linkage with PRN data (Figure 1). In the PRN database, 89702 deliveries were registered in the high incidence area during 2007 and 2008. Within this total population, 3095 singleton deliveries were excluded based on the indication that two or more had identical maternal date of birth and four-digit postal code. Thus, these deliveries belonged to different mothers and could not be validly linked to the serological data. None of the multiple deliveries with the same maternal date of birth and four-digit postal code originated from different mothers, as checked carefully by gestational age and number of foetuses. Of the 1678 eligible sera, 1169 (70%) could be linked to 1201 children registered in the PRN. For 25 children no neonatal outcome data were available and in two cases there was no clear laboratory result, thus a study population was generated composed of 1174 children who originated from 1155 mothers.

### Seroprevalence

The large majority (1646, 96%) of the 1713 sera that were available had been obtained from women living in 10 adjacent municipalities in the high incidence area. Based on the registered number of births in 2007, we estimated that during the study period 60% of pregnant women in these 10 municipalities were included in the study (Table 1). Of the 1646 sera, 40 had an IgM II and IgG II ≥1:64, of which four exhibited very intense fluorescence at the 1:64 dilution. In combination with the 16 sera with an IgM II ≥1:64 this gives an overall seroprevalence of 3.40%. Nineteen (1.15%) sera had anti-phase I and II IgG antibodies with a titre of ≥1:64 and without IgM being present (Table 1). For the linked mothers, the seroprevalence was found to be slightly lower, with 3.15% for possible recent infection (37 women, of whom 11 had solitary IgM II ≥1:64) and 1.02% for past infection. There was a clear correlation (Pearson correlation coefficient = 0.707) between seroprevalence among pregnant women and the incidence of notified symptomatic Q fever in the same municipalities. In addition to the 19 sera with anti-phase I and II IgG antibodies, there were 130 sera with a solitary IgG II ≥1:64. Out of the 1646 samples examined, 16 had an IgM I ≥1:64 and 30 had an IgG I ≥1:64. Although positive samples were not fully titrated, none of the IgG I positive sera exhibited intense fluorescence at the 1:64 dilution, making it unlikely that the titres would be very high and suspect for chronic Q fever.

**Table 1:** Presence of antibodies against Coxiella burnetii among pregnant women in 10 municipalities within the province of Noord Brabant, the Netherlands, from June 2007 to July 2008

Municipality	Pregnant women tested	IgG I and IgG II ≥1:64 (%)		IgM II and IgG II ≥1:64 or IgM II ≥1:64 (%)		Births in ** 2007**^**1**^	Q fever incidence ** in 2008**^**2**^
Oss	579	7	(1.2)	21	(3.6)	779	175
Uden	306	6	(2.0)	8	(2.6)	458	234
Veghel	293	3	(1.0)	5	(1.7)	377	38
Bernheze	240	1	(0.4)	15	(6.3)	326	344
Boekel	70	1	(1.4)	0	(0.0)	127	10
Landerd	60	0	(0.0)	4	(6.7)	122	791
Lith	37	0	(0.0)	3	(8.1)	57	342
Maasdonk	31	1	(3.2)	0	(0.0)	107	337
Sint-Oedenrode	15	0	(0.0)	0	(0.0)	151	23
Schijndel	15	0	(0.0)	0	(0.0)	219	17
Total	1646	19	(1.1)	56	(3.4)	2723	200

^1 ^Total number of births that were registered in each municipality in the year 2007

^2^Number of notified Q fever patients in the municipality from January 2008 to August 2008 per 100000 inhabitants (based on population size of the municipality on 1 January 2008)

### Pregnancy outcome

Average age at delivery was 30.8 years (standard deviation [SD] 4.2) for mothers with positive serology, and 30.5 (SD 4.5) for mothers with negative serology. The other characteristics of mothers and infants are shown in Table 2. There was a clear difference in overall seroprevalence by ethnic background (Dutch 2.6% *vs *non-Dutch 9.2%; *P *= 0.006). This difference was less pronounced when only positivity for anti-phase II IgM and anti-phase II IgG antibodies was considered (Dutch 2.9% *vs *non-Dutch 5.5%; *P *= 0.143). Babies from mothers with anti-phase II IgM and anti-phase II IgG antibodies were more often born in a non-vertex position, when only babies born after at least 37 weeks of gestation were considered (*P *= 0.012). For other characteristics of mothers and infants no statistically significant differences were observed between the groups with positive and negative serology.

**Table 2:** *Characteristics of mothers and infants and interpretation of serology for *Coxiella burnetii

Characteristic	IgG I and IgG II ≥1:64 (*n *= 12)		IgM II and IgG II ≥1:64 or IgM II ≥1:64 (*n *= 37)		No antibodies present (*n *= 1125)		**χ2 test**^**1**^
	
	*n*	(%)	*n*	(%)	*n*	(%)	*P*-value
Mothers							
*Parity*							
Primiparae	4	(33.3%)	19	(51.3%)	545	(48.4%)	
Multiparae	8	(66.7%)	18	(48.7%)	580	(51.6%)	0.836
*Ethnicity*							
Dutch	8	(66.7%)	31	(83.8%)	1021	(91.2%)	
Other	4	(33.3%)	6	(16.2%)	99	(8.8%)	0.006
Missing	0		0		5		
*Socio-economic status*							
Very high	0	(0%)	4	(10.8%)	134	(12.0%)	
High	1	(8.3%)	8	(21.6%)	171	(15.3%)	
Average	7	(58.3%)	17	(46.0%)	546	(48.7%)	
Low	3	(25.0%)	7	(18.9%)	199	(17.8%)	
Very low	1	(8.3%)	1	(2.7%)	71	(6.3%)	0.838
Missing	0		0		4		
*Urbanization (addresses per km*^*2*^*)*							
>1500	1	(8.3%)	6	(16.2%)	232	(20.7%)	
500-1500	6	(50.0%)	16	(43.2%)	481	(42.9%)	
<500	5	(41.7%)	15	(40.5%)	408	(36.4%)	0.538
Missing	0		0		4		
Infants							
*Gender*							
Male	6	(50.0%)	21	(56.8%)	553	(50.3%)	
Female	6	(50.0%)	16	(43.2%)	547	(49.7%)	0.508
Missing	0		0		25		
*Position*							
Vertex	12	(100%)	31	(83.8%)	1024	(93.4%)	
Breech or other	0	(0%)	6	(16.2%)	73	(6.6%)	0.131
Missing	0		0		28		
*Plurality*							
Singletons	12	(100%)	36	(97.3%)	1076	(95.6%)	
Multiples	0	(0%)	1	(2.7%)	49	(4.4%)	0.432

^1^Test for difference between antibodies present (columns 2 and 3) and absent (column 4)

Mean birth weight of the babies from mothers with positive serology was 3311 g (SD 637), while mothers with negative serology produced babies of 3354 g (SD 678). Presence of antibodies against *C. burnetii *was not significantly associated with any of the negative pregnancy outcome measures; the same was true when considering any adverse pregnancy outcome instead of each outcome separately (Table 3). Adjustment for confounders in multiple logistic regression analyses did not change the results (Table 4).

**Table 3:** *Pregnancy outcome by presence or absence of antibodies against *Coxiella burnetii

Outcome	IgG I and IgG II ≥1:64 (*n *= 12)		IgM II and IgG II ≥1:64 or IgM II ≥1:64 (*n *= 37)		No antibodies present (*n *= 1125)		**χ2 test**^**1**^
	
	*n*	(%)	*n*	(%)	*n*	(%)	*P*-value
*Perinatal mortality*							
No	12	(100%)	37	(100%)	1101	(97.9%)	
Yes	0	(0%)	0	(0%)	24	(2.1%)	0.151
*Apgar score*							
<7	0	(0%)	1	(2.7%)	22	(2.0%)	
≥7	12	(100%)	36	(97.3%)	1079	(98.0%)	0.983
Missing	0		0		24		
*Birth weight*							
<2500 g	2	(16.7%)	3	(8.1%)	86	(7.8%)	
≥2500 g	10	(83.3%)	34	(91.9%)	1015	(92.2%)	0.544
Missing	0		0		24		
*Birth weight by gestational age (<p10)*							
No	9	(75.0%)	35	(94.6%)	969	(89.0%)	
Yes	3	(25.0%)	2	(5.4%)	120	(11.0%)	0.858
Missing	0		0		36		
*Congenital malformations*							
No	12	(100%)	37	(100%)	1105	(98.2%)	
Yes	0	(0%)	0	(0%)	20	(1.8%)	0.346
*Gestational age*							
<37 weeks	1	(8.3%)	2	(5.4%)	109	(10.0%)	
≥37 weeks	11	(91.7%)	35	(94.6%)	987	(90.1%)	0.378
Missing	0		0		29		
*Any adverse outcome*^2^							
No	8	(66.7%)	32	(86.5%)	854	(75.9%)	
Yes	4	(33.3%)	5	(13.5%)	271	(24.1%)	0.358

^1^Test for differences between antibodies present (columns 2 and 3) and absent (column 4)

^2^Any adverse outcome: perinatal death, birth weight <2500 g, birth weight by gestational age <p10, Apgar score <7, or gestational age <37 weeks.

**Table 4:** *Multivariate analysis of seropositivity to *Coxiella burnetii *in relation to different adverse pregnancy outcomes*

Outcome	IgM II and IgG II ≥1:64 ** or IgM II ≥1:64**^**1**^		IgM II and IgG II ≥1:64 or IgM II ≥1:64 ** or IgG I and IgG II ≥1:64**^**2**^	
	
	**Adjusted OR**^**3**^	(95% CI)	**Adjusted OR**^**3**^	(95% CI)
Gestational age <37 weeks	0.7	(0.2-2.2)	0.9	(0.3-2.3)
Birth weight <2500 g	1.2	(0.4-3.4)	1.6	(0.6-3.9)
Birth weight by gestational age (<p10)	0.4	(0.1-1.8)	0.9	(0.3-2.3)
Apgar score <7	1.7	(0.4-8.2)	1.3	(0.3-5.6)
Any adverse outcome^4^	0.4	(0.2-1.2)	0.7	(0.3-1.5)

^1 ^Reference category: no antibodies present, and IgG I and IgG II ≥1:64 combined

^2^Reference category: no antibodies present

^3^OR was adjusted for maternal age, parity, plurality, ethnic background, socio-economic status, urbanization, and foetal position.

^4^Any adverse outcome: perinatal death, birth weight <2500 g, birth weight by gestational age <p10, Apgar score <7, or gestational age <37 weeks.

## Discussion

In our study population, presence of antibodies against *C. burnetii *in early pregnancy was not significantly associated with adverse pregnancy outcome. This is in contrast with published case reports and case series that suggest a high risk for adverse pregnancy outcome after an acute symptomatic or asymptomatic Q fever infection in pregnancy [4,16-18].

Any explanation for this discrepancy, such as possible differences in pathogenicity of different bacterial strains, is speculative as long as there are no data available. Reports from the literature on Q fever infection and pregnancy outcome have been limited by the fact that they have been based on small, selected numbers of pregnant women. However, the present study also has a number of limitations. The power of this study was negatively affected by the lower than expected seroprevalence and the relatively large number of women for whom a serum sample was available but ultimately could not be linked to the corresponding information in the PRN database. We were unable to determine the reason for record linkage failure in most of those cases. We made use of 12^th ^week sera, but only pregnancies with a gestational age ≥16 weeks were registered in the PRN; therefore, pregnancies ending between 12 and 16 weeks would have been missed. Thus, we cannot exclude the possibility that mothers with positive serology had higher rates of spontaneous abortion than did the mothers with negative serology. It is interesting to note, however, that antibodies suggesting recent infection were present in 4.0% of sera that were unable to be linked to the PRN, in contrast to 3.2% in sera that were able to be linked; this difference was not statistically significant (*P *= 0.376). The PRN registry contains no data on early pregnancy loss and spontaneous abortion, making this outcome measure unfeasible. During the study period, materials from 40 spontaneous abortions were investigated at the Jeroen Bosch Hospital but none of these materials was positive with PCR for *C. burnetii *(unpublished data).

It has been suggested that complications from acute Q fever occur more often when the infection takes place in early pregnancy [7]. However, there is a paucity of information on risks by trimester of infection. We were unable to provide data on women who might have seroconverted later (>12 weeks) in pregnancy. As no routine serum samples were available from late pregnancy, these data would be difficult to obtain and procuring such samples would present considerable logistical challenges, increased cost, ethical implications, and possible poor compliance of subjects. Some important risk factors for adverse pregnancy outcome, such as smoking during pregnancy, could not be taken into account. Heavy smoking during pregnancy is an item in the PRN, but has a low prevalence and is considered to be largely underreported. We had no information on occurrence of other prenatal infections, such as syphilis, *Listeria*, and group B streptococci, which are known to affect pregnancy outcome.

There is no consensus on serological methods and cut-off titres that should be used for population-based surveys in which only single titres are available; thus, it is difficult to compare results between different studies [19,20]. The largest study in the literature among pregnant women was carried out in south-eastern France, in which 12716 pregnant women were tested at the end of the pregnancy using an in-house developed IFA [21]. This study found a seroprevalence of 0.15% based on IgG II ≥1:100. Moreover, high prevalence areas had the highest proportion of preterm births, but the serological results could not be linked to pregnancy outcome at the individual level. In our study, 11 of the samples had an isolated IgM ≥1:64 against phase II. The other 26 samples had an IgM and IgG against phase II, both ≥1:64. A positive IgM against phase II has long been considered useful for the diagnosis of acute Q fever [22]. However, it has also been argued that an isolated positive IgM against phase II could be the result of an aspecific reaction. According to the manufacturer of Q fever IFA IgM, the diagnosis of recent *C. burnetii *infection requires a titre ≥1:16 for both phase I and phase II antigens (package inserts; Focus Diagnostics; 2009, http://www.focusdx.com/focus/packageInsert/IF0200M.pdf). Tissot Dupont showed that a combination of a phase II IgM titre ≥1:50 and phase II IgG titre ≥1:200 was 100% predictive for active Q fever [23]. It has been argued that local cut-off values need to be established, and for Denmark, a low prevalence country, a cut-off for the IgM phase II Focus Diagnostics IFA of ≥1:256 was suggested [24]. In the high incidence area of the Netherlands the prevalence of *C. burnetii *antibodies will be higher than those in Denmark and, therefore, the positive predictive value of a serological test will be higher at a lower cut-off. Furthermore, the choice of cut-off point, when there are no definite rules, must also depend on the reason for performing the test. In a screening programme a high cut-off would result in very few women with aspecific reactions being labelled as false positive. However, a number of women with recent infection but relatively low antibody titres would be missed. The cut-off chosen for the present study was a compromise between sensitivity and specificity of the screening test. In the context of the ongoing discussions on interpretation of serological methods, we have used 'presence of anti-phase II IgM and anti-phase II IgG antibodies' rather than the term 'recent infection' in the present study.

IgM phase II antibodies appear early in the disease and decrease quickly between weeks 10 and 15 [22,23]. The IgM response, however, may persist for a longer period of time and it is possible that women with a positive serology were infected just before pregnancy, which is probably less harmful than infection that occurs in early pregnancy.

The prevalence of anti-phase II IgM and anti-phase II IgG antibodies among pregnant women in this high incidence area is lower than expected. However, the study was carried out using samples obtained in 2007 and 2008, before the high incidence peak of Q fever in 2009. The relatively high seroprevalence among pregnant women with a non-Dutch ethnical background is consistent with findings from a nationwide seroprevalence study among the general population. A higher prevalence of past infection among people with a non-Dutch ethnical background was found, especially for individuals from Turkey [25].

The pathogenesis of adverse pregnancy outcome caused by *C. burnetii *infection is not fully understood. Infection of the placenta, placental insufficiency caused by immunological mechanisms, and direct effects on the foetus may all be contributing factors [4]. Similarly, the effects of acute Q fever during pregnancy on the newborn are unclear. In an endemic area in Egypt, 4 out of 100 cord sera obtained from normal newborns had high levels of IgM specific for *C. burnetii *[26]. In contrast, no evidence of foetal infection was found and cord blood taken at delivery was negative for IgM antibodies against *C. burnetii *in other case studies [27].

The large outbreaks of Q fever in the Netherlands have resulted in high awareness among patients and medical professionals. Laboratory tests are now widely available, including PCR-based tests that can provide an early diagnosis in the first two weeks of illness [28]. The ability for laboratory diagnosis of Q fever was much faster in 2008 and 2009, as compared to 2007 [29]. It can, therefore, be expected that most cases of symptomatic Q fever during pregnancy in the high incidence area will be detected and treated. Asymptomatic infections can only be identified by screening. Unfortunately, the present study cannot provide a clear answer to whether or not a large-scale screening program would provide benefits in terms of preventing adverse pregnancy outcomes and infection from occupational exposure.

## Conclusion

In conclusion, we found no evidence for adverse effects on pregnancy outcome among pregnant women with asymptomatic presence of anti-phase II IgM and anti-phase II IgG antibodies in early pregnancy. The present study provides insufficient basis for recommending large scale screening of pregnant women in high incidence areas. With the expanding Q fever problem, there remains an urgent need for a more definitive answer, and we anticipate that this will be provided by the large-scale prospective screening and treatment study which was initiated in March 2010. That on-going study is entitled "cost-effectiveness of a screening strategy for Q fever among pregnant women in risk areas: a clustered randomized controlled trial" and aims to include 4000 participants [30].

## Competing interests

The authors declare that they have no competing interests.

## Auhors' contributions

WvdH designed the study and drafted the study protocol with input from JCEM, ACAPL, DWN and CWPMH. JCEM was responsible for the laboratory analyses and NW organised the laboratory database. CWPMH and WvdH conducted the data analyses. WvdH drafted the final manuscript. All authors read and approved the final manuscript.

## Pre-publication history

The pre-publication history for this paper can be accessed here:

http://www.biomedcentral.com/1471-2334/11/44/prepub
